# 75 Years Ago: Discovery of Resin Adhesion to Acid-etched Enamel – A Comparison of the 1949 and 1955 Methods

**DOI:** 10.3290/j.jad.b5057135

**Published:** 2024-03-11

**Authors:** Hans Jörg Staehle, Caroline Sekundo

**Affiliations:** a Professor, Heidelberg University, Department of Conservative Dentistry, University Hospital Heidelberg, Heidelberg, Germany. Conceptualization (lead), project administration (lead), investigation (lead), original draft preparation (lead), reviewed and edited manuscript (equal).; b Assistant Professor, Heidelberg University, Department of Conservative Dentistry, University Hospital Heidelberg, Heidelberg, Germany. Investigation (supporting), wrote original draft (supporting), reviewed and edited the manuscript (equal).

**Keywords:** adhesion of acrylics to acid-etched enamel, history of adhesive dentistry, history of dental acrylics

## Abstract

**Purpose:**

This paper describes previously unknown details about the discovery of resin adhesion to acid-etched human enamel.

**Materials and Methods:**

A literature review was performed through manual assessments. Primary sources revealing the discovery of resin curing on etched enamel were analyzed considering the research objectives and methodological procedure during that era, including the type of teeth used, preparatory measures, acid-etching process, type of resin and its application, and follow-up observations. Additionally, the political and economic contexts were examined.

**Results:**

In 1949, acid etching was found to promote adhesion with acrylic resin, a finding described again in 1955. The 1949 studies utilized nitric acid for enamel etching and the acrylate resin Paladon from the Kulzer company (Germany). Conversely, the 1955 investigations employed phosphoric acid and an unnamed acrylate, likely a self-curing resin supported by Kulzer in the late 1930s. Disparities in the 1949 and 1955 findings can be ascribed to varying objectives and test conditions amidst a turbulent political backdrop, significantly impacting the Kulzer company.

**Conclusion:**

The discovery of resin adhesion to acid-etched enamel, approaching its 75th anniversary in 2024, is a landmark in 20th-century adhesive dentistry. Paladon represents a pioneering compound, exemplifying the influence of political, ideological, and economic factors on scientific advancements during that period.

The history of adhesive dentistry up to the mid-1950s was described by Staehle and Sekundo in 2021.^10^ This was followed in 2022 by a paper which, in addition to materials science and clinical aspects, also took into account political framework conditions (including health and professional policy).^11^

The two publications showed, among other things, that the phenomenon of adhesion of acrylic resins to acid-etched enamel was first discovered in 1949^9^ and independently investigated and described in 1955 as a possibility for clinical intervention (e.g., in the context of fissure sealants).^2^ However, little literature is available to date on the details of the technical procedure for the acid etching performed at that time and on the resins used.

In this paper, therefore, the methods of 1949 and 1955 are analyzed and compared. In the process, the resin compounds are also subjected to closer scrutiny. Finally, the accompanying political-ideological and economic circumstances to which the manufacturing aspect was exposed are also taken into account.

## Material and Methods

The primary sources, from which the objectives and procedures of 1949^9^ and 1955^2^ were evident, were evaluated with regard to the teeth used, the preparatory measures, the acid- etching procedures, the type of resins and their application, and the follow-up observations. On this basis, a comparative analysis was carried out. With regard to the acrylics used, the main focus was on the Paladon compound from the Kulzer company. In order to record the political-ideological and economic circumstances to which this company was exposed, conference papers of dental working groups, books, and articles on contemporary history as well as company portraits were reviewed.^3,5,6,8^ Biographical data on the discoverers in 1949 and 1955^4,7^ round off the subject matter.

## Results

### Presentation of the Two Approaches

#### 1949 Procedure (Method A)

The discovery of resin adhesion to acid-etched enamel in 1949 was an unintended chance observation.

In his dissertation on the remineralization behavior of dental enamel, which was accepted by the University of Tübingen (Germany) in 1949 and archived, the dentist Günter Staehle described investigations in which he artificially demineralized enamel surfaces by applying nitric acid and subsequently exposed them to the oral environment.

**Fig 1:**
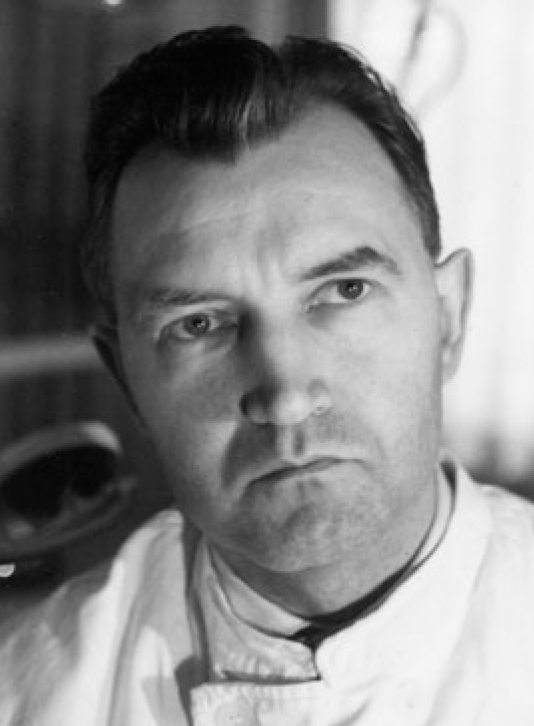
Günter Staehle (1921–2008) (year of photograph 1958). Source: Private archive H. J. Staehle.

Although the dissertation was not submitted until 1949, it is probable from the length of the procedure that at least some of the experiments described took place as early as 1948. There was no third-party funding for the study. The dentist G. Staehle (1921–2008; Fig 1) was a German citizen and worked most of his professional life in a practice in the city of Böblingen (Germany).^4^

Staehle made replicas for his analyses of tooth structures. He used human teeth for his studies, which he stored in physiological saline solution immediately after their extraction. After separating the roots of the teeth, he mounted the remaining tooth crowns in dentures worn by patients. What number and types of teeth he used is not clear from his dissertation paper. He did not perform any cleaning of the tooth surfaces, but only superficial drying. In order to achieve a defined enamel etching, he placed plastilin on the enamel surfaces to be examined, in each of which he made a circular recess of 1 mm^2^. After exposure to 5% nitric acid for 3 minutes, he detached the plastilin and rinsed with water. After drying, he found a white, matte surface in the etched area. For replica fabrication, he applied the liquid of Paladon acrylic resin from the Kulzer company to the tooth surface and waited until a thin film was formed by solidification about 1 to 2 minutes later. The film proved to be crystal clear, stable and sharp. He then pressed a cellophane strip onto the solidified Paladon film. He removed the strip together with the acrylate film from the tooth surface. He observed variable adhesion behavior. In some cases, there was no adhesion. In other cases, however, adhesion was so strong that the acrylate film could not be removed. He interpreted this adhesion as a consequence of the surface roughness of the etched enamel. He wrote about this in his dissertation: “Various other difficulties in detaching the film arose later, especially in etched areas, whose roughness favors adhesion to the tooth surface.” However, he found no plausible reasons for the different adhesion behavior. He pointed out that a few days after exposure of the study teeth to the oral cavity, the white and dull enamel surfaces had disappeared and there was no longer any difference in color.

Through his observations, he was thus not only the first to discover the adhesion of acrylate-based resins to etched enamel, but also correctly interpreted the increase in adhesion as a physical (micromechanical) rather than a chemical process.

However, he did not realize the significance of his discovery, namely that the adhesion improvement between enamel and acrylic resin caused by acid etching could be useful for many dental purposes. Rather, the adhesion enhancement between acrylic and etched enamel was a methodological problem for him, which complicated his experiments. He did not see it as a promising opportunity for other applications.^9^

#### 1955 Procedure (Method B)

Unlike the situation in 1949, the description of resin adhesion to acid-etched enamel in 1955 was the result of targeted research.

**Fig 2:**
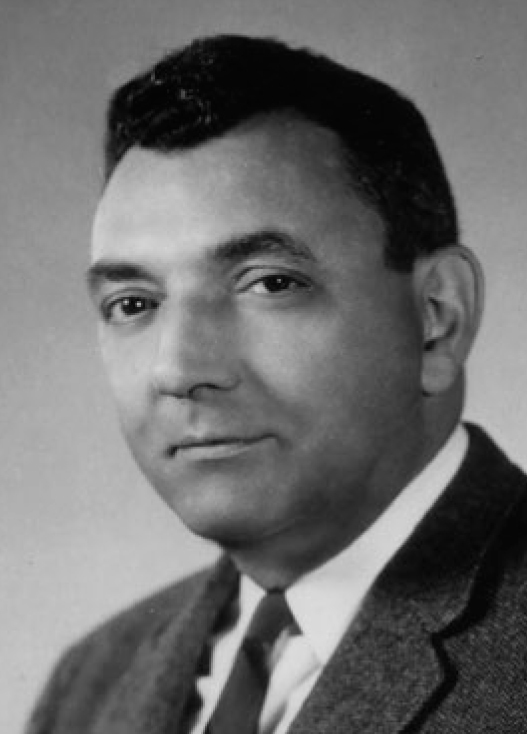
Michael G. Buonocore (1918–1981) (year of photograph 1953). Used with permission from the Edward G. Miner Library, University of Rochester, Rochester, N.Y.

The chemist and dentist Michael G. Buonocore (1918–1981; Fig 2) was looking for ways to make acrylic adhere to the tooth surface in order to open up clinically relevant areas of application (e.g., fissure sealants to prevent caries).

Buonocore was an American citizen with Italian roots who worked in various fields of the military (US Army dentist at Fort Knox, Kentucky/USA) and university (Eastman Dental Center, Rochester, NY/USA).^7^ The paper was submitted on July 14, 1954, about 3 months after the start of the trial. In this respect, both the experimental performance and the writing of the manuscript took place in 1954. It was funded by US Army appropriations (supported by the Medical Research and Development Board, Office of the Surgeon General, Department of the Army, under contract No. DA-49-007-MD-330).^2^

Buonocore experimented with various material combinations (phosphomolybdate in combination with oxalic acid as well as phosphoric acid). Etching with phosphoric acid followed by the application of acrylic resins proved to be the most successful.

After preliminary tests on extracted teeth, he used in-situ teeth of volunteer subjects for his main tests, mainly maxillary and mandibular incisors, “occasionally” also premolars and molars. More detailed information on tooth types and shapes is not given in his publication. Before etching, he cleaned the teeth with pumice and alcohol. After drying the tooth surface, he applied 85% phosphoric acid for 30 seconds, followed by water rinsing. He mixed acrylic resin according to the manufacturer’s instructions (but without declaring the name of the material or the manufacturer) and applied a drop of resin with a diameter of about 5 mm^2^ to the etched area. It is not clear from the publication whether or not he took measures to ensure the size of the area mentioned, and if so, which.

. He waited for the resin to harden and then smoothed its surface. He then observed whether the resin fell off by itself or had to be separated mechanically. In the latter case, he used “considerable force with a sharp instrument,” but without describing this in detail. He tested the stability of the adhesive behavior by observing the retention time of the resin applied to the teeth, distinguishing between untreated and etched teeth (n = 10 each). For the untreated teeth, adhesion lasted an average of 11 hours until the specimens fell off on their own. In contrast, for the etched teeth, the average adhesion was 1070 hours (= 45 days). In half of the treated teeth (5 teeth), the resin was removed instrumentally (as described above), sometimes resulting in cohesive fractures within the resin. The other half (also 5 teeth), at the time of writing his manuscript (90 days after the start of the trial), still had the acrylic overlays, which resisted detachment even with “strong thumbnail force.” He interpreted the adhesion as a purely physical phenomenon. In the case where the resin had been mechanically detached, the enamel surface initially appeared opaque and white, only to return to its original appearance after a few days.

#### Comparison of Methods A and B

The two methods described in 1949 and 1955 show strong differences (Table 1). While the adhesion of acrylic resin to etched enamel observed in 1949 was classified as an undesirable effect that hindered the realization of an experimental study, the adhesion described in 1955 was a desired result. The adhesion results of the 1949 study varied more widely than was the case in the 1955 study. No reason for the wide variation was found at that time. A comparison of Methods A (1949) and B (1955) provides several explanations:

**Table 1:** Resin adhesion to etched enamel with comparison of Method A (1949) and Method B (1955)

No.	Description	Method A (1949)	Method B (1955)
1	Dental substrate	Extracted human teeth (without assignment to specific tooth types). Immediately after extraction, the teeth were placed in physiological saline solution, followed by separation of the tooth roots and mounting of the remaining tooth crowns in patient-worn prostheses	In-situ human teeth (maxillary and mandibular incisors, occasionally also premolars and molars)
2	Cleaning of the tooth surfaces	No cleaning measures described	Cleaning with pumice and alcohol
3	Surface drying before etching	Yes	Yes
4	Size of the enamel area to be etched	1 mm^2^ (first covering the enamel surface with plastilin, into which a circular recess of 1 mm^2^ was made for the defined acid application)	Initially not exactly defined; later, a drop of acrylate with a diameter of approx. 5 mm^2^ was applied to the etched area (see below)
5	Melt etching	5% nitric acid	85% phosphoric acid
6	Etching time	3 minutes (followed by detachment of the plastilin)	30 seconds
7	Rinsing after enamel etching	With water	With water
8	Surface of the etched area after drying	White and matte	Initially not described (cf. no. 14)
9	Material used	Paladon liquid (Kulzer)	Acrylic filling resin mixed according to manufacturer’s instructions (without indication of preparation name and manufacturer)
10	Application	Application of Paladon liquid to the etched and unetched enamel surface	Application of a drop of resin with a diameter of approximately 5 mm^2^ to the etched area (without information on how the size of the intended adhesive area was realized)
12	Application time, post-treatment	1-2 minutes, until a hard film was formed by means of solidification	Waiting until the resin hardened, then smoothing the resin surface
13	Separation from the tooth surface	Pressing a cellophane strip onto the solidified Paladon film with subsequent removal	Waiting for the resin to fall off by itself or removing it with a sharp instrument
14	Adhesion	Variable: sometimes no adhesion, sometimes adhesion so strong that detachment was not possible; adhesion was interpreted as a consequence of the surface roughness of the etched enamel (physical adhesion)	A comparison was made between untreated and etched teeth; in the untreated teeth, adhesion averaged 11 hours; in the etched teeth, it was 1070 hours (= 45 days); adhesion was interpreted as a physical phenomenon
15	Follow-up	A few days after wearing the study teeth, the white and matte surface in the etched area disappeared	After resin detachment, the enamel surface was initially opaque and white but returned to its original appearance after a few days

- In Method A, in contrast to Method B, the tooth surfaces had not been cleaned before etching.
- In Method A, etching was performed with 5% nitric acid, which apparently did not produce as effective an etching pattern as 85% phosphoric acid.
- In Method A, the adhesion area was only 1 mm^2^, while in Method B it was approximately 5 mm^2^.
- The thin resin film of Paladon prosthetic material used in Method A probably had a lower polymerization rate and strength after solidification than the self-curing resin in Method B, which was applied in a much thicker layer.

Common to both methods was that human teeth were studied, which – despite the use of different acids – showed a uniform etching pattern (white and opaque surface structure after drying) that disappeared again when exposed to the conditions of the oral cavity. The most important common feature was that an adhesion effect had occurred despite highly different experimental conditions.

### Differentiation of the Acrylate Resins Used

#### Dental aspects

##### Method A

The Paladon compound used in Method A was the first acrylic resin ever found to exhibit adhesion to etched enamel.

Acrylic resins were introduced into dentistry in 1930 by the chemist Walter Bauer (who was employed by the Darmstadt-based company Röhm & Haas AG). Initially, they were sold as precured compounds that could be molded under heat and pressure (a process called “dry processing”). In 1936, the “dry” processing technique was replaced by the “wet process,” invented by the dental technician Gottfried Roth. In this process, the polymer powder polymethyl methacrylate (PMMA) was mixed with the monomer liquid methyl methacrylate (MMA). The resulting plastically deformable masses were pressed into a mold and cured in boiling water (“hot polymerization”). This process was patented by the Kulzer company in 1936 and introduced into dentistry under the brand names Paladon (for dentures) and Palapont (for crowns and bridges). It became apparent that the liquid MMA tended to polymerize even when exposed to light or weak heating, i.e., it cured “itself” in a certain way, although very slowly, which is why stabilizers were later added to it. This may have been the reason why, in the 1949 tests, the applied Paladon spontaneously solidified into a thin film. However, polymerization did not proceed uniformly, which, in addition to the omission of tooth surface cleaning, makes the variable bonding behavior understandable (see Section “Comparison of Methods A and B”, above).

##### Method B

The material of the resin used in Method B was not described in the 1955 study. It only mentions an acrylic resin that was mixed according to the manufacturer’s instructions. It can be assumed that this was a self-curing resin of the type that had been available since the late 1940s.^10^ Resins of this type were developed in the second half of the 1930s by the craftsman dentist Ernst Schnebel and patented in 1940. Schnebel also collaborated with the Kulzer company, which had the new materials tested in animal experiments, among other things.^10^

#### Political-ideological and economic aspects

##### Method A

Paladon was used primarily as a prosthetic resin to replace the rubber that had been common until then. At the time, the manufacturing Kulzer company was based in Frankfurt am Main.

In June 1938, Friedrich Schoenbeck, head of the chemical-metallurgical laboratory and professor at the Dental University Institute in Berlin, gave an overview on the subject of “Synthetic resins as dental materials” at a conference of the working groups for prosthetics and materials science of the German Society of Dentistry and Oral Medicine (DGZMK). This paper was published in 1939. Schoenbeck wrote that synthetic resin fulfills the “demand of Generalfeldmarschall Göring in that we are dealing here with a material which not only has essentially the same properties as the material to be replaced, but which surpasses it by far”.^8^

Regarding the compound Paladon, he made the following statement: “About one of these materials I must say a few words here, namely about the Paladon. Paladon is a useful substance, but since it is produced by Jews, the state institutes are forbidden to use this Paladon clinically in any way, and rightly so. We have made various attempts to exclude the Jews, and the Reichsstelle, with which we cooperate, has always been informed by us accordingly. However, we have not yet been able to count on a purely Aryan company. However, such a company will now be formed. Until this will have happened*, the situation is such that we can, of course, talk about the Paladon here in our circle as much as we want, but that we must keep a low profile in public”. The note in the footnote* read, “The difficulties have now been solved, nothing stands in the way of processing the Paladon in practice. (The Editor)”.^8^

In 2004, the historian Peter Hayes described how the “difficulties” mentioned by Schoenbeck were solved.^5^ He dealt with “Aryanization measures” by companies such as Degussa or Heraeus. Hayes noted that the company Degussa worked to legally appropriate “stolen goods” in “Aryanizations.” He wrote: “The vigorously pursued takeover of Kulzer & Co. Frankfurt, the ninth takeover of an industrial enterprise from ‘Jewish’ hands altogether, was a ‘forced Aryanization’ in the more radical sense, because the Gauleiter of Hesse decided on the sale and the three Jews (Messrs. Frank, Fuld, and Isaacson^1^) among the four owners played virtually no role in the negotiations. Kulzer manufactured high-quality dentures and the like, mainly from a resin-based material called Paladon, which had been developed by the fourth partner, an ‘Aryan’ and Swiss citizen named Gottfried Roth, based on patents of Röhm & Haas AG in Darmstadt [...]. In addition, the Ministry of Economics put pressure on Röhm & Haas to supply only ‘Aryan’ molds. Since a large part of Kulzer’s products were sold abroad and thus brought in much needed foreign currency, the rapid ‘Aryanization’ of Kulzer was of great importance for the local and national economy...”. By 1938, “the shares and net profits were divided between Degussa and the Heraeus company.” According to Hayes, the Kulzer owners were coerced into a “forced sale” to Degussa and Heraeus for less than their value, becoming “deeply involved in a naked robbery”. After World War II, Degussa and Heraeus apparently got off lightly in repayments to heirs beginning in 1950, though a “complicated banking transaction” caught the attention of the Wiesbaden Chief Finance Office and prompted an inquiry. Hayes noted that “Degussa successfully defended the transaction, pointing out that this left Kulzer in German ownership; the company would have been lost if the heirs had recovered their 75% shareholding”. Hayes noted that from an economic point of view, “a balance sheet of the ‘Aryanizations’ continued to be positive well into the postwar period”.^5^

In 2016, Gramm et al suggested why the Heraeus company had been predestined for the takeover of Kulzer also for political reasons. The director of the company Heraeus, Wilhelm Heinrich Heraeus had set up a National Socialist Business Cell (NSBO) in his factory at an early stage and had publicly declared himself to be an “ardent Hitler supporter”.^3^

In a report entitled “Backstage at Kulzer: A Hessian success story” from 2017, the processes are described as follows: “The success story begins in 1935 in Frankfurt am Main: Franz Kulzer founds Kulzer & Co. GmbH with his partners Jacob Frank and Arthur Fuld. Initially, the company sells an impression compound made of synthetic resin and denture material in plate form made of Cellon. Already in the following year, it develops the first heat-polymerizable resin Paladon 65. With this invention, Kulzer does pioneer work. Thanks to the new prosthetic resin, the previously common and ill-fitting rubber prostheses could be replaced. Paladon – in an improved form – is still part of Kulzer’s dental technology portfolio today. More than 75 years ago, the companies Heraeus and Degussa finally took over Kulzer in equal parts. It was not until 1987 that Heraeus Holding GmbH became the sole owner of Kulzer GmbH and founded the subsidiary ‘Heraeus Kulzer’ in 1995”.^6^ There is nothing about the career of the name-giver Franz Kulzer in these and the other quoted explanations.

In summary, it can be stated that Paladon, which was patented by the Kulzer company in 1936 during a politically highly charged period and used for the experiments in 1949, came from a manufacturer that had been owned equally by the Heraeus and Degussa companies since 1939 and is now in the sole possession of Heraeus Holding GmbH.

Taking into account the current state of knowledge, the name Paladon not only stands as a pioneering compound for dental prosthodontics but also for adhesive dentistry. Furthermore, it bears exemplary witness to oppressive, political-ideological and economically motivated arbitrary measures in the 20th century.

##### Method B

The inventor of self-curing resins, Ernst Schnebel, was the head of the “Main Testing Laboratory” of the Reich Association of German Dentists (RDD). As a vocationally-trained dentist, he did not have an easy position vis-à-vis the academic dental profession. As late as 1941, Friedrich Schönbeck, the university professor quoted above, felt compelled to relativize the significance of Schnebel’s activities and to place them in the vicinity of “conscious propaganda”. He wrote that he recognized Schnebel’s “certainly very laborious work” but did not necessarily want to call it a “scientific deed”. Schnebel’s research results became internationally known only through the so-called Blumenthal Report of 1947, a pronouncement of the Office of Military Government for Germany U.S., the highest administrative institution of the American occupation zone of Germany. This report, entitled “Recent German Developments in the field of Dental Resins (field information agency, technical united states group control council for Germany; abbreviated F.I.A.T.),” provides information on the development of the self-curing resin.^1^ According to this report, Blumenthal visited Degussa (Frankfurt) and its “subsidiary” Kulzer (Friedrichsort im Taunus) in military-occupied Germany after the Second World War and obtained information from the management there. In his report, he pointed out that the production of the first self-hardening acrylate (Palapont S. H.) was based on the discovery of E. Schnebel. Regarding the early sources on this from 1939 onwards, he commented as follows: “Very little has been published on the subject except for the information contained in war time patent applications and the patents granted to Kulzer & Co on the basis of those applications (cf. D.R.P. applications D. 85578-IV c/39 C, 7/29/41; French 88, 3679, 3/19/43; Swiss G 74, 466, 7/25/42; Swedish 3.896, 6/7/42; etc.). The product has never been manufactured except on an experimental scale”. Shortly after the publication of the Blumenthal Report with its disclosures, numerous such products were offered on the market worldwide, e.g., in 1949 the preparation “Rapid-Palodont” by the Degussa and Heraeus subsidiary Kulzer (for details see Staehle and Sekundo, 2022^10^).

It can therefore be assumed that acrylate resins based on earlier research by Kulzer were also used for the tests published in 1955.

## Discussion

Although the 1949 and 1955 experiments were completely different in their objectives and procedures, they ultimately led to the same result, namely, that acid etching of tooth enamel can improve resin adhesion.

In one case (1949), the phenomenon was undesirable for the researcher as it hindered his studies;^9^ in the other case (1955), it was welcomed by the researcher, as it provided an opportunity to come closer to achieving his goals.^2^

Both studies exhibited significant shortcomings in the presentation of their methodologies. For instance, the 1949 study failed to detail the number and types of teeth examined or the quantified limitation and verification of the adhesive behavior (1949). On the other hand, the 1955 study lacked crucial information such as the type of resin used and the preparation of a defined bonding surface, among other things.

Because of these limitations, the research results could hardly be published according to today’s standards. Nevertheless, they led to epoch-making discoveries, with the groundbreaking work of 1955 now considered a “classic”, ranking among the most notable publications in dentistry.^7^

These facts hold remarkable significance, not solely from a dental historical perspective, but also in understanding the history of science.

From a political-ideological and economic standpoint, the results shed light on the attempts during the 20th century to sideline the manufacturer of a pioneering material for political, racist, and economic reasons, aiming to overshadow its products. When these attempts failed, the owners were coerced into a sale that was unfavorable to them. Even though later reparation payments were made, the transaction proved to be advantageous for the buyers at the time, reflecting positively on their overall balance sheet and ultimately contributing to a unique “success story.”

## Footnotes

1 *Jacob Frank, Arthur Fuld, Arthur Isaacson (he later changed his name to Saxon).

## Clinical relevance:

This review of resin adhesion to acid-etched enamel highlights a key development in adhesive dentistry, emphasizing the influence of historical context on the evolution of dental materials and practices.
